# Guidelines for Using Simulation for Online Teaching and Learning of Clinical Social Work Practice in the Time of COVID

**DOI:** 10.1007/s10615-021-00807-x

**Published:** 2021-05-03

**Authors:** Uschi Bay, Marcelo Maghidman, Jacinta Waugh, Aron Shlonsky

**Affiliations:** Department of Social Work, Monash University, Melbourne, Australia

**Keywords:** Social Work education, Simulation, Clinical Practice Laboratory, Distance education, Coronavirus

## Abstract

Due to COVID 19, Monash University’s Social Work Department moved all clinical practice skills teaching in the Master of Social Work (graduate entry level) fully online using synchronous audio-visual conferencing platform Zoom for the first time from March to June 2020. The innovations associated with this move included the development of clinical practice laboratories (CPLs) to prepare 154 students for a modified version of an Objective Structured Clinical Examinations (OSCE) and their first field education practicum. The use of simulated clients to facilitate experiential learning of active listening skills, rapport-building and empathic communication in this mode of delivery is described in detail to encourage overcoming previous issues in teaching clinical practice skills to students located at a distance from campus.

## Introduction

The Department of Social Work at Monash University was an Australian pioneer in the delivery of distance education (DE) to its off-campus Bachelor of Social Work students in 1989(Oliaro & Trotter, 2010; Afrouz and Crisp 2021). Over thirty years ago, the internet as we know did not exist and our approach to off-campus teaching was to provide print material for each course, including topic readings and self-reflection questions. Students, though, were required to attend on-campus, intensive skills-based learning modules for several days each year. Though it seems almost quaint at this point, email was touted as the new technology that would facilitate student and staff interaction for most of the year (Oliaro & Trotter, 2010). The introduction of distance education modes in social work programs was very controversial due to the concerns about whether the acquisition of clinical practice skills and other requisite social work attributes could be achieved without regular face-to-face contact. This same controversy continues (Jones, 2015), though objections appear to be, in the main, ideological in nature given that substantial evidence exists for the efficacy of DE for skills-based training (Afrouz and Crisp 2021).

Despite these concerns, there has been continued growth of distance education offerings across the world and, as of 2020, there are 16 DE Social Work programs accredited by the Australian Association of Social Workers (AASW). However, the AASW continues to reinforce its specific requirement for students undertaking these programs to attend on-campus training for a minimum of 20 days throughout their degree to demonstrate their social work skills in person (face-to-face) with teaching staff. Regardless of the arguments for this mode of delivery and the concerns about its efficacy for training social workers in clinical practice skills, there is an increasing number of social work programs being delivered online across the world including, amongst many others, countries like Canada, USA, UK, India, Israel and Norway (Oliaro & Trotter, 2010).

When the COVID-19 pandemic hit Australia in 2020 and Melbourne went into ‘lockdown’,^1^ Monash was able to quickly move all of its students into an online delivery format. The timing of the pandemic fortunately coincided with technological advances that have made internet access common for most households and the availability of video communication a regular practice for a large proportion of the population. The availability of internet technology has enabled a replication of lectures, now audio-visually recorded and/or streamed; and participation in tutorials and small groups for teaching clinical skills is now possible on Zoom.^2^ We quickly moved all clinical skills training online for *all students* whether enrolled in on-campus or off-campus study modes during this world health crisis.

## Simulations in Social Work Clinical Practice

Objective Structured Clinical Examination (OSCEs) were first developed for assessing clinical competencies in social work students by Marion Bogo and Colleagues at the University of Toronto, Canada and Azusa-Pacific University in California (Bogo et al. 2014) and has since made its way as far south as Australia and New Zealand. The use of OSCEs at Monash with Master of Social Work (Qualifying) students, began in 2019. The Master of Social Work comprised of students who do not have an Undergraduate Bachelor of Social Work hence achieving clinical competencies are an essential part of this degree. Successful completion of the Master of Social Work enables students to be eligible for membership of the AASW and to enter the workforce as a graduate social worker. The introduction of client simulations in the format of a clinical practice laboratory (CPLs) occurred in 2020.The pedagogical notions informing our adoption of these approaches are in line with the holistic model of competence presented by Bogo et al. (2006). This model identifies meta-competencies and procedural competencies (Bogo et al. 2012). Meta-competencies relate to students’ cognitive capacities, knowledge, values, alertness to interpersonal interactions, self-awareness, self-reflection and self-regulation of emotions. Procedural competencies comprise skills and techniques that prepare students for interviews, assessments, interventions and communication (verbal and non-verbal) in social work practice. They focus on the ability of first year Master of Social Work students developing a collaborative relationship with the client, to undertake an assessment, collaboratively set goals with the client and develop an ever-increasing sensitivity to cultural and structural issues impacting on the client.

Along with the University of Toronto approach, a major influence on the development of the clinical practice laboratories was to create a ‘community of practice’ amongst the students, instructors and actors (Lave & Wenger, 1991). To facilitate the development of clinical skills and build trust among students undertaking the modified OSCE assessments three clinical practice laboratory (CPLs) sessions where held with students in small groups (maximum of 16 students). It was hoped that the (CPLs) would decrease anxiety prior to each students’ required demonstration of skills in the modified OSCE (McDermott, 2002). Further, based on social learning theory, we surmised that students would learn from observing their peers conducting a live interview with a simulated client, one who could embody a carefully developed client scenario (Asakura et al. 2020; Bandura, 1977; Stegmann et al. 2012). Indeed, the vicarious learning possible through students observing each other for an additional 6 h also enabled the three instructors to reinforce students’ engagement with the relevant meta-competencies, focusing considerable attention on case conceptualisation.

## Lessons from Conducting OSCEs Pre-COVID 19

In 2019, after conducting the modified OSCE assessment the three main instructors observed that a number of international students, in particular, found it difficult to identify the client’s presenting problem, the precipitant event (what brought the client along that day) and were struggling to paraphrase content and reflect the client’s feelings. Many of the international students were overly reliant on asking questions to engage the client, and offered rather formulaic responses to client’s feelings, with standard phrases like “that sounds tough” or “that must be hard”. Students, instead of using the client’s own words (motifs, metaphors) and really naming or identifying the intensity of the emotions expressed by the client, were not clear on how to respond to the client's feelings and content. Further, it seemed difficult for students to accurately reflect the exact level of emotions expressed by the client with the result that the client’s feelings were either minimised or exaggerated. This meant that the client did not feel they were correctly understood or were accurately listened to by the student (Geldard et al. 2017). It also inhibited, for some students, their ability to express the empathy or unconditional regard they were meant to be offering to the simulated client. Apart from the possible language issues for some of the international students, awareness of the role of clinical skills in social work practice was potentially another aspect we needed to address. One of the ways of addressing these difficulties was to model social workers engaging with simulated clients in various settings and to demonstrate the use of clinical skills in direct social work practice. The three instructors planned to provide three CPL sessions in 2020 to address these difficulties and to provide these to all students in the course. Due to COVID this meant introducing this innovation on Zoom. The Clinical Practice Laboratories (CPLs) were conducted in three separate sessions for two hours each, over a period of two months prior to the students undertaking the modified OSCEs.

## Teaching Clinical Practice Laboratories fully Online

In 2020, 154 students enrolled in the course (SWM5102) a first-year course in the Masters of Social Work and with 83 students (more than 50%) identifying as having English as an additional language, the main instructors hoped the three CPLs would assist with scaffolding students' clinical skills acquisition. The idea of a practice laboratory was partly inspired by University of Toronto’s “Practice Fridays” (Kourgiantakis et al. 2019). The CPLs consisted of 3 two-hour Zoom meetings with up to 16 students each, hence overall the three instructors conducted 28 CPLs during the first semester in 2020. Each CPL used a different client scenario with the simulated client maintaining their character for the duration of the lab. This allowed students to interact sequentially across each two-hour laboratory session with the simulated client. The three CPLs were specifically focused on:

1. Identifying the presenting problem and precipitant event, conducting the beginnings of a case conceptualisation, and the use of specific micro skills for information gathering (e.g., use of open and closed questions, clarification and summarising).
2. Responding to the client using active listening, paraphrasing, and other relationship building/ engagement strategies; beginning use of metaphor and interpretation of client meaning.
3. Responding to the client’s expressed feelings in the interview including reflecting the expressed emotion at the appropriate level and not minimising or exaggerating the intensity of the client’s feelings.

The first CPL was dedicated to assisting students to understand the presenting problems, their precipitant event(s), understand the client’s current situation, and identify the main issues affecting the client. The second CPL focused on paraphrasing (reflection of content) to allow student time to practice this skill; the overuse and abuse of questions was challenged and alternative ways of responding to the simulated client was provided to the students (Geldard et al. 2017) and students were given the opportunity to rehearse these within the laboratory sessions. The third and final CPL session concentrated on reflection of feelings, tuning into client’s specific emotional expression and intensity level of emotions. The aim was for instructors to reinforce and show the interrelationship between the use of micro-skills and the demonstration of empathy with the simulated client. One of the challenges the instructors faced was the inability of many students to recognise and accurately name the simulated client’s emotions. Enlarging students’ vocabulary and recognition of emotions including some of the nuances and intensities was particularly relevant in this exercise. Students were also asked to consider any cultural differences in the way emotion is expressed. Many assumptions were challenged, with the aim of practicing ways of reflecting emotion accurately and without judgement in the service of building the therapeutic alliance (Geldard et al. 2017; Prout & Wadkins, 2014) (Table 1).

The structure of the CPL sessions allowed the instructors to pause the session to discuss, answer questions, demonstrate skills, and establish the direction to be taken next in the client interview by students.

The ability of the simulated clients to remain in character and hold a cohesive storyline for the students with ‘real’ emotional expression added to the student’s engagement with the simulated client over the two hours timeframe. Each student would interact with the simulated client for up to 9 min. If needed the student could be interrupted or could interrupt the interview to gain input or support from their peers or instructor. The instructor was able to encourage students to try another approach to asking a question- perhaps rephrasing a question to reflect a strength based rather than deficit approach to a situation, if required. This was considered a crucial advantage of the CPLs as in previous years students at times learned approaches to interviewing clients that did not work and students found it difficult to unlearn later what they had learned in their peer to peer role plays. The CPLs aimed to prevent this from occurring by immediately encouraging students to change their approach and to try out and test another approach (e.g., a strengths-based approach) and to observe the impact of the change in their approach on the client. The instructor would then invite the next student to continue the same session from where the previous student left off. After finishing their section of the interview, feedback was offered to the student interviewer from the instructor and simulated client, as well as some of their student peers. Students also reflected on their learning at the end of the CPL session by outlining what they had learned from the session, what they would take away with them from their experience in the CPL and to identify which micro-skill they would practice for the rest of the week. According to Slovák et al. (2015) it can be comforting for students to learn by observing while also being challenged to genuinely interact with the simulated client without prior knowledge of their backstory.

## Scenarios for Micro-Skill Development

The actors were provided with the scenarios containing information about each client’s current circumstances, background and emotional responses to their situation prior to each Clinical Practice Laboratory. All actors were trained in the Howard Fine method (www.howardfine.studio.com.au) which meant that the actors tapped into their own emotional reactions and thus presented an emotionally convincing simulated client. Our previous experience with using some actors not trained in this method was that they conveyed the content of the scenario well to the students but did not emote in a way that assisted students to engage with the emotions evident in the interview. Hence, it was difficult for students to reflect the simulated clients’ feelings and this made student paraphrasing of both content and reflection of feeling harder to demonstrate (Geldard et al., 2017). The advantage of the actors embodying the emotions in the interactions also indicated how the simulated client’s communication patterns likely maintained the client’s current difficulties in their situation. The actors were able to do this effectively as this was foundational to their specific training, making interactions very realistic and enabling students to genuinely engage with the client and their situation.

## Training of Simulated Clients

The actors were trained to participate in three different types of simulations throughout the semester in this course (SWM5102). The first was a recording of a simulated client interview conducted by one of the three instructors with the purpose of students watching the video to undertake a biopsychosocial assessment. In this type of interview, the simulated clients were providing a great deal of information without much prompting from the social work interviewer. The students were tasked with gaining information and content about the client’s story, the use of previous services by the client, dynamics in the client’s family of origin, any medical or mental health concerns, types, frequency and levels of social connections, client’s current living situation, employment and educational achievements. The second type of simulation was the CPL where the simulated client was required to be in “character” for two hours while various students and the instructors interviewed them as a social worker. The simulated clients were briefed on the specific setting and the set of issues and concerns as these related to social work as outlined in the prepared scenario. The third type of simulation was the modified OSCE which required each actor to be familiar with 16 different client scenarios and to be interviewed for 10 min at a time by a student social worker in two hours Zoom session conducted over a four-week period (Table 2).

The simulated clients were progressively introduced to each of these types of simulations, the purpose of the simulation and the learning expected by the students was clearly explained. The simulated clients asked many questions around the intensity of the emotions to be exhibited and the willingness to disclose more layered aspects of the client’s feelings and backstory. The simulated clients were encouraged to be realistic but also sensitive to students’ abilities. For instance, simulated clients’ reactions to students who were not listening and or asked the same questions in different ways were to be realistic. These reactions were unpacked in the CPLs by the instructors to illustrate the effect of the students’ attitude, body language, line of questioning on the client. The simulated clients were asked to move on from their negative reactions to the students interview after a pause in the OSCE’s. The pause was to mark the place where the student was not engaging the client and to enable the student to reflect on their skills and the impact of these on the simulated client when writing up their critical reflection of the clinical skills they exhibited during their OSCE.

The simulated clients were proactive in facilitating the students different learning needs within each type of simulation. This assisted the students' learning and ability to demonstrate their skills. For example, in the modified OSCE interview the simulated client did not offer as much information at the start of the interview but waited for the student to inquire into their situation, and similarly responded to students’ different approaches in the CPL while staying in “character” often with surprising results. Student interviewers, at times, pursued a hunch or assumption that did not hold for the client. The simulated client’s response could then surprise the student, highlighting the impact of an assumption emanating from the student’s line of questioning, it also made obvious when students used loaded questions to guide the interview. Moreover, this illustrated how a student interviewer’s hunch could detract from their ability to remain actively listening with the client. The professionalism of the simulated clients and their desire to assist the students to become effective social workers were commendable, and the students appreciated the work of the simulated clients by often thanking them for their work.

### Scenario Scripts

The following is an example of one of the scenarios given to the simulated actors in preparing themselves for student interviews. The scenario scripts were designed for either a male or female simulated client. These script outlines prompted simulated clients to engage their emotions and to create a convincing backstory to convincingly portray the client to the students.

### Simone

Simone is referred to the social worker at the community health service by her General Practitioner (GP). The GP is concerned that Simone is showing signs of high anxiety. Simone was diagnosed with HIV only a week ago and her GP is worried that Simone is not coping with this news. It appears Simone has asked twice to be retested to make sure the diagnosis is accurate. The GP has confirmed that there is no doubt about the diagnosis. The GP is concerned that Simone may harm herself or her partner.

Simone in the interview alternates between exhibiting a high state of anxiety and a despondent attitude. She is unable to believe she has contracted HIV. She is very upset;/she has worked out that she has contracted the disease from Abdullah.

Simone fell in love with Abdullah 18 months ago. Abdullah moved in with Simone two weeks after they met at a friend’s garden party. Abdullah had migrated from Saudi Arabia and was working at a company that sold earphones. Simone could not believe her luck. Abdullah was handsome, some years younger than her, charming and reciprocated Simone’s affection. Abdullah was generous with his affection, and sweetly thoughtful towards Simone. Abdullah also had a wicked sense of humour and lightened Simone’s angst and more anxious moods. Simone had never been happier and felt her life had finally fallen into place. Abdullah was everything she wanted in a man and more.

Previously, they had spent most evenings together, had dinner together and gone out together to see shows, dance, visit friends and been almost inseparable. More recently, Simone recounts that Abdullah had started to go out more on his own. Abdullah had suggested that they needed to have a little bit more space in their relationship and started to go out for after work drinks with his colleagues.

Abdullah has quickly moved out of the apartment with Simone and is not returning her calls.

The GP at the community health centre was aware that a few years prior Simone had been self-harming after an event where she had lost her job due to a complaint by a colleague that she had been sexually inappropriate towards her. Simone took this situation very hard and went into a major depression. The GP at that time referred Simone to a psychiatrist who prescribed anti-depressants and also worked with Simone using various therapeutic approaches. Simone found the combination helpful and she also gained some insights about the dynamics operating in her family of origin. Simone says she learned from the therapy that she has “father issues”. Simone recalls that the ‘therapy’ was helpful to her in that she felt less anxious and depressed afterwards.

This scenario was one of the many used as the basis for a two hours CPL where the instructors modelled the initial 15 to 20 min of the interview with the simulated client Simone in the role of the community health social worker. This modelling was to enable students to observe the interaction between the client and the social worker live on Zoom and to ensure the students gained some initial sense of the situation Simone was facing.

The CPL also allowed the CPL instructors (who were also the academic Unit Coordinators and lecturers in the unit) to answer students’ questions raised by the scenarios about direct social work practice. The CPL enabled the facilitators to pause the interview and call attention to various behaviours by the client and by the student conducting the interview. It was possible to highlight on Zoom the student’s own and client’s body-language and the impact of the types of questions used on the interaction. The CPL instructors were able to assist with reframing students’ statements or questions towards more supportive and strengths-based language. These sessions also allowed students to rehearse these reconfigured attempts at phrasing their questions anew. Students were also given time to practice identifying the emotions expressed by the client and in reflecting these back to clients as accurately as possible. The simulated client's ability to resume the interview, stay in “character” and continue expressing various emotional states made this a very rich learning experience for students. The CPL format also meant the instructor could focus students’ thinking on their meta-cognition of the client’s situation and emotions, drawing links to various theories. For example, psychodynamic theory could be applied to the case of Simone by helping students to identify repeating behavioural patterns (Coady and Lehmann 2018).

## Outcomes for Students

Outcomes for students in their performance on the OSCEs in 2019, which was offered in a face-to-face format, were similar for students in 2020 which was offered solely on Zoom. There is reportedly little difference between on and off campus students’ performance in social work education, according to Afrouz and Crisp (2021). Specifically, off campus students can perform as well, in relation to their overall field competency and clinical skills, compared to their counterparts on campus (Afrouz and Crisp 2021). In this instance, where *all* students were taught clinical skills in synchronous interaction on Zoom sessions, there was little difference in performance detected between the two cohorts. The differences that were noted were influenced by other more pertinent factors: one, the recent arrival of some students in Australia; and two, students’ experience of holding conversations primarily in English who were not used to doing so. Indeed, Levine et al. (2013) discussed that these students would need to be brave to speak in an online environment as everyone is listening to them. It was critical for CPL instructors to take a coaching role that was supportive for all students and encouraged each one to engage and trust their contribution would be welcomed as a learning opportunity. Although there is a lack of literature related to this specific difficulty for international students, Levine, for whom English is not a first language and is relating personal experiences as a lecturer, suggests that the online environment is very challenging for non-English speakers (Levine et al. 2013).

The qualitative feedback from unit evaluations completed anonymously by students were, overall, very positive and clear with respect to the important role of the CPLs in assisting them to prepare for the OSCEs. Most students also expressed that they wanted even more opportunities to practice their interpersonal interviewing skill using simulated clients. Further, many students mentioned how much they enjoyed the interaction and engagement with the other students in their Zoom group. Many stated they found the weekly required attendance helped them to stay engaged in the course and they valued the frequency and quality of the synchronous interaction with instructors and their peers. Both distance education and on campus students valued the practice interviews with simulated clients and the helpful feedback from the instructors after each section of the interview.

## Discussion

In 2020, we conducted 176 modified OSCE’s online (22 students needed to repeat their OSCE, 12 passing on their second attempt) with a simulated client using Zoom to assess their preparedness for field education placement. To support students with the OSCE assessment task, a series of three CPLs were developed to facilitate students’ capacity to conduct a client interview using the video-conferencing platform Zoom. The CPL format allowed social work instructors to model micro-skills or interpersonal skills to all students synchronously, and to encourage individual students to try out their clinical skills with simulated clients before undertaking their OSCE and working with actual clients in their field practicum. The CPL format then gave students the opportunity to rehearse appropriate responses such as paraphrasing of content and reflecting of feelings disclosed by the simulated client. This seemed to work well as all students were able to observe, comment, interact and test out their skills with the support of the instructor and peers in an immediate interchange (Jones, 2015). The CPL also allowed students to observe efficient clinical practice skills, as modelled by the instructors, and they could ask questions about these interactions (rather than simply watching a video of an unrelated expert practitioner working with an unknown client). Additionally, students were also able to identify and discuss the development of their meta-cognitive skills by linking theory with practice, discussing their use of self, and by observing the impact of their own and others’ use of micro-skills with the simulated client (Biggs, 2012).

As outcomes for students in their performance on the OSCEs in 2020 were not different to the precious year, this suggests that students learning in a synchronous way with personalised interactions with their instructors may be one of the vital components to obtaining these outcomes (Noble & Russell, 2013). It appears that some of the online limitations outlined by social work educators previously (Goldingay and Land 2014) were overcome by the synchronous teaching on Zoom. It may be that a new definition of face-to-face teaching may be required for social work education and the related accreditation bodies with the development of technologies like Zoom (Smith et al. 2018). Further, familiarity with Zoom technology prepares social work students for social work practice that may be provided using audio-visual conferencing technology and potentially for field education placements that also use telehealth approaches.

This experience has prompted us to consider a number of ways to improve our delivery of online CPLs and OSCEs. These include:

- using more socio-culturally diverse actors in the simulated case scenarios. In 2019 and 2020 we only used Caucasian simulated clients, a wider range of actors is required to reflect the diversity of clients using social work services in Australia.
- setting up informal drop-in times for students before and after CPLs to encourage the type of discussion between instructors and students that would normally occur prior to and after an on-campus class. We think this can go a long way toward normalising the online experience and gives students the opportunity to quickly deal with smaller but still important matters that might otherwise be ignored, and to build relationships with their instructors.
- scheduling a formal debriefing session one week after each CPL to capture students’ learning and reinforce it, obtain feedback, and engage with students about changes that can be made to facilitate further learning.

The relevance of past disputes about whether teaching “interpersonal relationships requires face-to-face, real time interaction” (Jones, 2015, p. 230) now seem distant. COVID-19 forced us to find a way to deliver high-quality social work training in an online format, and our observations suggest that frequent and quality synchronous online interaction may achieve similar outcomes. The CPL allowed for synchronous instruction with a simulated client where the instructor was able to offer a classroom like experience. This innovation required additional resources and time by instructors in order to facilitate the increase in intensity and frequency of interaction with students. The simulated clients needed more preparation for a larger volume of scenarios to increase the diversity of interview experiences. CPL and tutorial times also needed to be scheduled in the evening to meet all students needs including part-time, Distance Education students and off shore international students in different time zones across the globe.

Unfortunately, we were not able to compare the different outcomes between on and off campus students in 2020 as all of our teaching was fully online. Further research into the effectiveness of online simulated interviews and synchronous video-conferencing platforms for teaching clinical social work skills is clearly needed. Our observations are an important first step but represent a fairly low standard of evidence of effectiveness. In the near term, an additional step worth considering is to explore whether the clinical practice skills gained through the CPLs and OSCEs translate into better interviewing skills and experiences when students are faced with interviewing real clients, in person, as part of their field education practicum.

**Table 1:** Summary of steps taken in the Clinical Practice Laboratory Sessions

Clinical Practice Laboratory (CPL) Sessions	CPL Session 1	CPL Session 2	CPL Session 3
Who starts the interview	Instructor interviews simulated client for 15–20 min	Student volunteer engages the simulated client	Student volunteer engages the simulated client
Skills being shown and practiced	Role clarification Negotiates confidentiality Establishes purpose of the interview and presenting problem Demonstrates micro-skills Paraphrases client’s content and feelings	Role clarification Confidentiality Clarifies purpose of the interview- referral information Aims to identify precipitant event and presenting problem Active listening Paraphrase client’s content	Role clarification Negotiates confidentiality Clarifies purpose of the interview- referral information Aims to identify precipitant event and presenting problem Active listening Paraphrase client’s content Reflect client’s feeling and content
How and what students practice	Various students continue the interview with the simulated client in up to 5–6 min sections focusing on open and closed questions, clarification, attending behaviour	Students continue the interview with the simulated client in up to 5–6 min sections focusing on paraphrasing of content	Students continue the interview with the simulated client in up to 5–6 min sections focusing on paraphrasing and reflection of feelings
Instructor feedback	Instructor provides feedback to each student	Instructor provides feedback to each student and interviews simulated client for 10–12 min	Instructor interjects during students' interviews and provides feedback

**Table 2:** The three different types of simulations for actors

Type of simulation	Description of simulation	How and when simulation was used
Biopsychosocial assessment	Pre-recorded session interviewed by one of the instructors filmed at the beginning of the semester for 20 min	A different interview was shown to each tutorial class for the student’s in-class biopsychosocial test
Clinical Practice Laboratory (CPL)	Scenarios for interview by “one” social worker who is comprised of the instructor and the 16 students in the Zoom session for two hours each	Each CPL had a different scenario There were three scenarios for each actor to learn
Modified OSCEs	Four 10-min interviews conducted by student social worker in each small Zoom class for four weeks	Each student had a separate scenario Actors needed to learn at least 16 scenarios

## Footnotes

1 In March 2020, Victoria began what turned out to be an almost continuous 6-month shutdown of all schools and non-essential businesses, with the population required to stay in their own homes except for shopping, caring responsibilities and exercise.

2 That said, some students may be disadvantaged if access to technology and the internet is not available and affordable. In our experience, however, we had limited technical issues and these were resolved fairly quickly.
